# Revealing Sex Differences During Upper and Lower Extremity Neuromuscular Fatigue in Older Adults Through a Neuroergonomics Approach

**DOI:** 10.3389/fnrgo.2021.663368

**Published:** 2021-08-16

**Authors:** Ranjana K. Mehta, Joohyun Rhee

**Affiliations:** ^1^Wm. Michael Barnes '64 Department of Industrial & Systems Engineering, Texas A&M University, College Station, TX, United States; ^2^Department of Environmental and Occupational Health, Texas A&M University, College Station, TX, United States

**Keywords:** fNIRS, gender, motor variability, hand grip, knee extension

## Abstract

**Background:** Sex differences in neuromuscular fatigue is well-documented, however the underlying mechanisms remain understudied, particularly for the aging population.

**Objective:** This study investigated sex differences in fatigability of the upper and lower extremity of older adults using a neuroergonomics approach.

**Methods:** Thirty community-dwelling older adults (65 years or older; 15 M, 15 F) performed intermittent submaximal fatiguing handgrip and knee extension exercises until voluntary exhaustion on separate days. Muscle activity from prime muscles of the hand/arm and knee extensors were monitored using electromyography, neural activity from the frontal, motor, and sensory areas were monitored using functional near infrared spectroscopy, and force output were obtained.

**Results:** While older males were stronger than females across both muscle groups, they exhibited longer endurance times and greater strength loss during knee extension exercises. These lower extremity findings were associated with greater force complexity over time and concomitant increase in left motor and right sensory motor regions. While fatigability during handgrip exercises was comparable across sexes, older females exhibited concurrent increases in the activation of the ipsilateral motor regions over time.

**Discussion:** We identified differences in the underlying central neural strategies adopted by males and females in maintaining downstream motor outputs during handgrip fatigue that were not evident with traditional ergonomics measures. Additionally, enhanced neural activation in males during knee exercises that accompanied longer time to exhaustion point to potential rehabilitation/exercise strategies to improve neuromotor outcomes in more fatigable older adults.

## Introduction

Neuromuscular fatigue, defined as the susceptibility to exercise-induced loss of strength, can disrupt intended motor behavior and exacerbate functional declines in older adults (Berrios, 1990; Hunter, 2016). These may include functional deficits, such as inability to reach and grasp objects or climb stairs, increased incidents of falls, and mortality rates (Lewis and Wessely, 1992; Tralongo et al., 2003; Mehta and Agnew, 2008; de Rekeneire et al., 2014). Neuromuscular fatigue has shown to differ between men and women, and this difference is task-dependent (i.e., intensity, muscle group used, contraction mode; (Hunter, 2014, 2016). Fatigue during isometric contractions, that do not involve any joint movement, have shown large sex differences. While women have shown to be less fatigable than men, this is largely observed with more postural muscles, such as the elbow flexors and back and knee extensors, than the smaller distal muscles of the ankle (Hunter and Enoka, 2001; Hunter et al., 2006, 2009). Moreover, sex differences diminish during high intensity when compared to low intensity fatigue tests (Maughan et al., 1986; Yoon et al., 2007).

Because sex differences in fatigability is task-specific, the underlying mechanism(s), i.e., central and/or peripheral, can differ based on the attributes of the fatiguing task. Peripheral mechanisms explaining sex differences in fatigability have been studied extensively (reviewed in Hunter, 2014) and have been a major focus in ergonomics (Iridiastadi and Nussbaum, 2006; Mantooth et al., 2018; Abdel-Malek et al., 2020). For instance, during sustained isometric contractions at low-to-moderate relative intensities, sex differences in skeletal muscle physiology and perfusion can explain greater fatigability in men than women, owing to restricted blood flow and increased intramuscular pressure (Hunter and Enoka, 2001; Mantooth et al., 2018). However, during intermittent isometric contractions, that facilitates muscle perfusion (Hunter et al., 2009), it is likely that central fatigue plays a key, if not dominant, role (Taylor and Gandevia, 2008). For example, women exhibit increased central fatigue development when compared to men during lower extremity isometric contractions (Martin and Rattey, 2007), and across 3 to 30 s contraction periods (Clark et al., 2005; Ansdell et al., 2017), however these differences have shown to attenuate during upper extremity fatiguing contractions (Hunter et al., 2006).

Sex differences during fatiguing exercises of the lower extremity muscles have been previously reflected in compensatory changes in rate of motor unit activation, measured using surface electromyography (Clark et al., 2005; Ansdell et al., 2017) or force variability, i.e., fluctuations measured using coefficient of variation and complexity measured using entropy analyses, of resulting force output (Svendsen and Madeleine, 2010). However, both males and females are reported to consistently exhibit greater muscle activity during upper than lower extremity exertions, indicating that muscle activation strategies may vary more between muscle groups than between sexes (Avin et al., 2010). Similarly, entropy measures of low back (Sung et al., 2008) and elbow (Svendsen and Madeleine, 2010) muscle fatigue have shown to capture sex-dependent force control mechanisms, implicating increased musculoskeletal risk in women owing to lower force variability. Unfortunately, the lack of systematic evaluation of sex differences in neuromuscular strategies during fatigue manifestation of different muscle groups have hindered any progress on determining fatigue mechanisms that are sex- and muscle-group dependent.

Several imaging studies have reported sex differences in brain structure (e.g., relative brain volume, cortical thickness, gray matter; (Good et al., 2001; Sacher et al., 2013) and connectivity (Ingalhalikar et al., 2014) that may potentially influence central mechanisms of fatigue. During an upper extremity finger tapping motor task of comparable performance, women exhibited increased activation of ipsilateral and bilateral cortex than men (Lissek et al., 2007). However, during force control of ankle dorsiflexion muscles, (Yoon et al., 2014) reported no sex differences in brain activation indicating comparable ability of men and women to activate cortical motor centers during isometric contractions. It should be noted that these imaging studies did not focus on fatigue development. When motor tasks are performed to voluntary exhaustion, which stresses the corticomotor pathways, it is likely that sex differences in neural activation may emerge. However, prior studies that examined brain regions during motor fatigue failed to examine and/or report sex differences (Dai et al., 2001; van Duinen et al., 2008; Keisker et al., 2009; Fry et al., 2017). It is important to determine sex differences in the recruitment of key brain areas associated with motor fatigue of different muscle groups in older adults. Such investigations will yield a better understanding of central control mechanisms of motor impairments and functional declines in men and women, which may inform research that addresses and enhances age- and fitness-related plasticity to preserve physical functioning.

Little is known regarding sex differences in brain activation during neuromuscular fatigue of muscle groups that differ in structure and function. Due to physical and functional constraints of the existing brain imaging techniques, there is limited work focused on determining brain activation patterns during neuromuscular performance of large postural lower extremity muscles (e.g., quadriceps). Published studies that measure brain activation during knee movement have focused on adaptations post injuries or medical events (Grooms et al., 2015). Quadriceps muscle performance plays a major role in maintaining functional mobility and falls prevention in the geriatric population (Singh et al., 2010). Functional near infrared spectroscopy (fNIRS), an optical brain imaging technique, can offer to fill this gap since it allows for mobile neuroimaging during both upper and lower extremity neuromuscular tasks (Quaresima and Ferrari, 2016). fNIRS, a popular neuroergonomics tool (Zhu et al., 2020), also provides relatively good spatial and temporal resolution to measure task related brain activation changes in different motor-function related cortical regions.

The purpose of this study was to investigate sex differences in spatiotemporal neural activation changes during upper and lower extremity isometric fatiguing contractions in older adults using a neuroergonomics approach. Muscle activity from prime muscles of the hand/arm and knee extensors were monitored using electromyography (EMG) and force output were obtained during intermittent isometric fatiguing handgrip and knee extension exercises at 30% maximal voluntary contraction (MVC). Neural activity of the frontal and sensory/motor (S1/M1) areas of both hemispheres were monitored using functional near infrared spectroscopy (fNIRS) to investigate the fatigue related changes in cortical areas. These areas were selected as they are known to be related to exertions of the upper and lower extremity musculatures (Mehta and Shortz, 2014; Sukal-Moulton et al., 2014). We hypothesized that the sex differences in fatigability will be muscle-dependent, and that these differences will be associated with differences in spatiotemporal neural patterns.

## Methods

### Participants

A convenience sample of 30 older adults (15 males, 15 females) were recruited from the local community. All participants were 65 years or older, right-hand dominant, and reported to be sedentary to recreationally active individuals who did not have any musculoskeletal injuries or disorders (e.g., Carpel Tunnel Syndrome, arthritis) that would interfere with the study protocols within the past year. All participants were provided financial compensation upon completion of their participation. The Institutional Review Board at Texas A&M University approved the procedures and participants provided written informed consent before data collection. Detailed participant demographic information is provided in Table 1.

**Table 1:** Participant demographics.

	**Male**	**Female**	***p*-value**
Age (yrs.)	74.3 (6.2)	72.3 (4.9)	0.33
Height (cm)	177.7 (7.8)	163.6 (5.8)	<0.001^*^
Weight (kg)	77.4 (8.6)	60.7 (6.9)	<0.001^*^
BMI (kg/m^2^)	24.4 (1.2)	22.7 (1.9)	0.006^*^
Physical Activity (# of steps/day)	7553 (2368)	7726 (2907)	0.86

*Values are presented as Mean(SD)*.

* *Indicates significant sex differences (p < 0.05)*.

### Procedure

Participants attended three sessions on different days: one preliminary and two experimental sessions. During the preliminary session, participants' physical characteristics including, height, and weight were obtained. At the end of the preliminary session, a 3-axis accelerometer (activPAL^e^, Pal Technologies Ltd, UK) was attached to participant's thigh to measure physical activity (PA). Participants wore this sensor continuously for 7 days and the sensor was received back from the participants at the start of the first experimental session, which occurred one week after the preliminary session. The recorded physical activity was reported as average number of steps per day.

**Figure 1:**
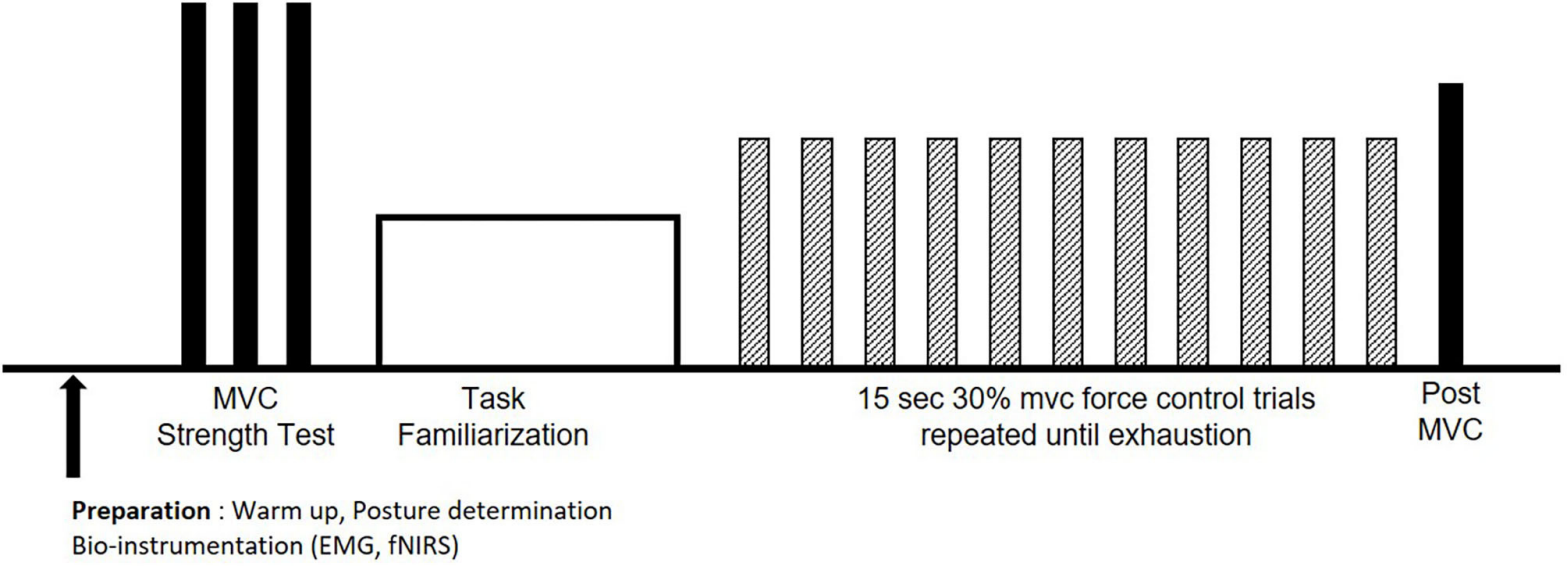
Experimental procedures for each session. After warm up, bioinstrumentation, and posture determination, maximum voluntary contractions (MVCs; solid bars) were performed, followed by task familiarization. Participants performed the endurance test at 30% MVC (hatched bars) until voluntary exhaustion, followed by a post MVC (solid bar).

At each experimental session, participants underwent intermittent isometric fatiguing handgrip, on one day, and knee extension exercises, on the other day. The sessions were separated by at least 48 h and the order of the two sessions were counterbalanced across the study sample to minimize fatigue carryover effects. Procedures for each session were similar (Figure 1): warm up, participant posture determination, bioinstrumentation, strength testing, task familiarization and practice, endurance testing, and final post strength testing. Posture determination for the two sessions were as follows. For the handgrip session, participants were seated upright on an isokinetic dynamometer chair (Humac, Computer Sports Medicine, Stoughton, MA), their dominant upper ar m placed at their side, elbow flexed at 90°, and lower arm in a neutral posture supported on an armrest. For the knee extension session, participants were seated upright in the same isokinetic dynamometer chair with hip and knee flexed at 90°, and their upper body secured firmly in the chair to minimize upper body motion caused by lower extremity muscle contractions. The dynamometer's center-of-rotation was aligned with that of the right knee joint and then locked with a dynamometer pad secured above the ankle, anterior to the tibia. Participants were then instrumented with surface electromyography (EMG) electrodes on the respective muscles (based on the experimental session; please see section Electromyography) and functional near infrared spectroscopy (fNIRS) head cap and probes (described later in Section fNIRS).

Before any strength testing, participants were provided 2-min warm ups; gripping/relaxing a stress ball at the handgrip session, and intermittent pushing against the dynamometer pad at the knee extension session. During the handgrip strength testing, participants performed isometric maximum voluntary contractions (MVCs) by maximally gripping a hand dynamometer (BIOPAC, CA, USA) for 3 s. During the knee extension strength testing, participants performed isometric MVCs by maximally extending their lower leg against the dynamometer pad for 3 s. For each testing day, three MVCs were measured with 2 min of rest in between each MVC trial. For each participant, the maximum of the three repeated MVCs determined the initial strength for the handgrip and knee extension session. Additionally, the MVC values were used to determine the 30% MVC level for each participant's endurance test for that session. Real-time visual feedback and verbal encouragement were provided during the MVC trials.

The experimental task required participants to perform intermittent submaximal force control trials at 30% of their MVC, determined during the strength testing, for 15 s followed by 15 s rest. A 440 Hz audio tone with 500 ms duration was presented to participants at the beginning of each contraction and rest period to indicate the contraction period. The 30% MVC target force level was presented as a red line on a computer screen at eye height and participants were instructed to track their generated force against the target level as closely as possible. Participants familiarized themselves with the experimental task by performing 3–5 trials. More practice was provided if the participants were not able to control their exerted force around the target line. After adequate rest (~5 min), endurance testing began. Participants were instructed to perform the intermittent force control trials as precisely and for as long as they could, until voluntary exhaustion. Verbal encouragement was provided during the endurance task. The task ended when either the participant voluntarily discontinued the exercise or their exerted force/moment dropped >10% below the required effort level for more than 3 s. Immediately after the endurance task ended, a post-MVC was measured to quantify strength loss based on the MVC value obtained at the start of that session. Participants provided Ratings of Perceived Exertion (RPEs) after each force control trial (Borg et al., 1987).

### Measurements

Handgrip force and EMG signals were collected at 1,000 Hz (Biopac Inc., Ca, USA) during the entire handgrip exercise session. Knee extension torque was collected with 100 Hz sampling rate (Humac, Computer Sports Medicine, Stoughton, MA). Neural hemodynamic responses were recorded using a continuous wave fNIRS system with a sampling rate of 50 Hz (Techen Inc. MA, USA, CW6 system) during the entire session. The collected signals were pre-processed for analysis using Matlab R2016a (Mathworks, MA, USA). The concurrent multi-modal data stream, collected from multiple devices, were synchronized using the audio signal provided to participants as event stimulus. The same audio signal was transmitted concurrently to the other devices dedicated for data collection of muscle activation (EMG) and neural hemodynamic responses (fNIRS) for data synchronization. During the analysis, measurements were synchronized by synchronizing the temporal location of the audio signals recorded in each device.

#### Strength, Fatigue, and Perceived Exertion

The initial and post-MVC values for each session were used to calculate strength loss (calculated as [initial-post]/initial MVC) for that session. Endurance time was determined at task cessation; either when the participant's force level dropped below 90% of target force level for longer than 3 s or when the participant voluntary terminated the task. RPEs were collected after every force control trial using a 10-point scale, ranging from 0 “*Nothing at all*” to 10 “*Extremely strong, almost maximum*” (Borg et al., 1987).

#### Force Variability

Force/torque data were low-pass filtered at 15 Hz, and middle 12 s of data in each force control trial was extracted for force variability analysis. Coefficient of variation (CV) of force for 1 s window in each trial was calculated and then averaged across each trial to assess force steadiness (Enoka et al., 2003). The complexity of force time series data was obtained by calculating the sample entropy (SaEn) of each force control trial, which provide a measure of the apparent randomness or regularity of a system's output (Richman and Moorman, 2000). SaEn was computed as the conditional probability of the signal by providing a measure of the (logarithmic) likelihood that runs of patterns that are close for *m* embedding dimensions remain close for incremental (*m* + 1) comparisons. The false nearest neighbor approach was applied to determine the embedding dimensions used in the present study *m* = 4 (Kennel et al., 1992). The tolerance distance parameter r that indicates r times the standard deviation of the segment in the time series was determined as *r* = 0.2 (Samani et al., 2015). Higher SaEn values (total range of 0–2) indicates lower predictability of future data points and greater irregularity in the force time series data, while lower values represent greater repeatability and regularity. Neuromuscular fatigue has shown to reduce SaEn (Pethick et al., 2019).

#### Electromyography

EMG signals of the extensor and flexor carpi radialis (ECR, FCR, respectively) muscles were recorded during the handgrip exercise, and the signals of the Vastus Lateralis and Rectus Femoris (VL, RF, respectively) muscles were recorded during the knee extension exercise. Recorded signals were band-pass filtered (20–450 Hz), rectified, and detrended. Root mean squared (EMG RMS) value of middle 12 s window of each trial was calculated (Srinivasan et al., 2016), and normalized to the EMG RMS value during the initial MVC test. Mean RMS of EMG is a crude indicator of neural effort (Farina et al., 2010), and while it has some limitations, it provides for a simpler approach compared to EMG decomposition methods (Farina et al., 2010), and thus was used to capture changes in neural drive changed over time.

#### fNIRS

**Figure 2:**
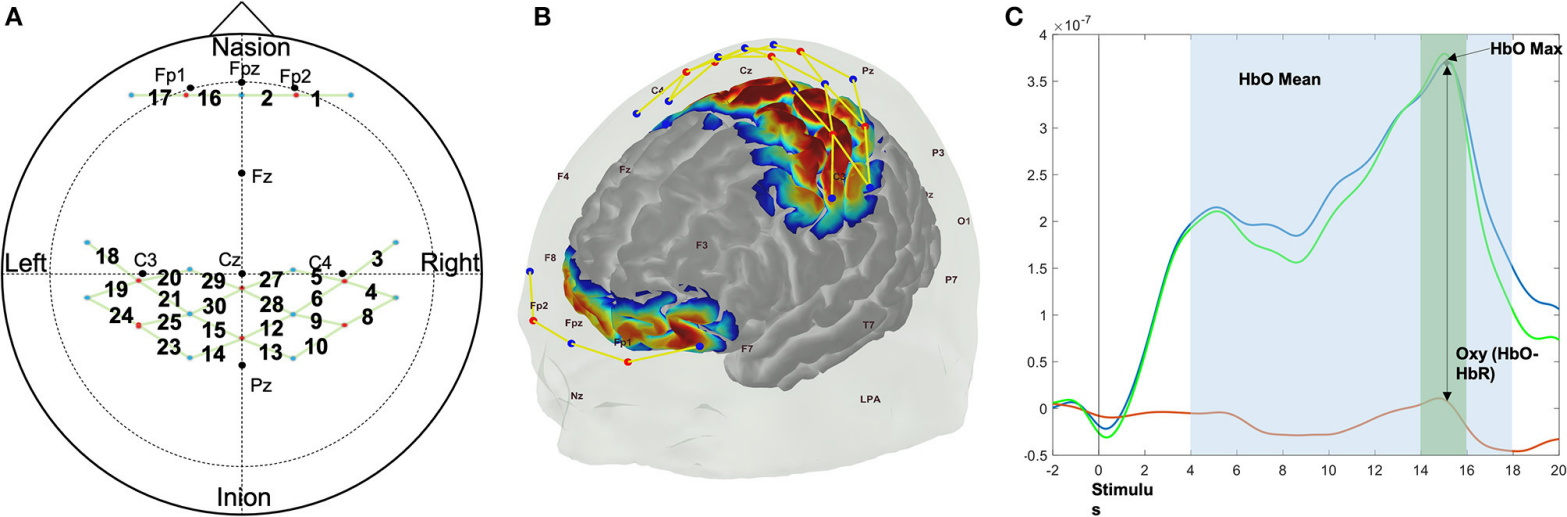
fNIRS custom probe design **(A)**, the sensitivity map on the brain surface that shows the areas of interest (frontal, motor, and sensory area) measured using the custom probe design **(B)**, and the sample hemodynamic responses during force control trials that shows how neural activations are quantified in the present study **(C)**. The red dots show the location of light emitters and the blue dots show the location of detectors. The yellow lines between red and blue dots show the location of each channel (numbers) where the hemodynamic responses were measured **(A,B)**.

The anterior prefrontal cortex (PFC), primary motor area, and sensory area were monitored across 26 channels through a custom NIRS probe, using the 10–20 International System, consisting of eight emitters and 13 detectors (Figures 2A,B). Recorded hemodynamic responses were preprocessed using HomER2 (Center for Functional Neuroimaging Technologies, Massachusetts General Hospital East, MA; (Karim et al., 2012). The light intensity was converted into optical density changes and low pass filtered with a 3 Hz cutoff frequency to reduce high-frequency noise. The motion artifact caused by the sudden head movement was corrected using the kurtosis based wavelet algorithm (Chiarelli et al., 2015) after the extreme signal offsets were corrected using spline interpolation algorithm (Scholkmann et al., 2010). Post evaluation and correction of the motion artifacts, the optical density signal was band-pass filtered with a 0.5 Hz to 0.016 Hz cutoff frequency range to remove physiological noise caused by heartbeat, and possible slow wave drifts caused by NIRS system (Koenraadt et al., 2014). Processed optical density changes were then converted into hemodynamic responses changes of Oxygenated (ΔHbO) and Deoxygenated (ΔHbR) hemoglobin concentration using modified Beer-Lambert law. The average ΔHbO and ΔHbR levels of 2 s window prior to event stimulation of each trial was subtracted from the average ΔHbO and ΔHbR levels of 2 s around maximum ΔHbO and ΔHbR levels within 4−18 s during each force control trial to acquire task-related neural hemodynamic response changes (Figure 2C) (Mehta and Rhee, 2017; Rhee and Mehta, 2019). Statistical analyses were conducted on ΔHbO as it has shown greater sensitivity to task related changes compared to ΔHbR (Malonek and Grinvald, 1996).

### Statistical Analysis

Independent sample *t*-tests were used to test sex differences in the demographic data and physical activity collected during the preliminary session. Initial strength, strength loss, and endurance time for both handgrip and knee extension sessions were also submitted to independent sample *t*-test to examine any sex differences. For each muscle-specific fatiguing session, RPEs, force variability, EMG, and fNIRS measures were averaged within three phases normalized to each participant's endurance time and labeled as early, middle, and late fatigue phases. Separate mixed-factor analyses of variance (ANOVAs) were performed to test the effects of sex (male vs. female) and fatigue phase (early vs. middle vs. late) on RPE, CV of force, SaEn of force, EMG RMS, and each channel of the ΔHbO for each muscle group. Statistical significance was determined at alpha level of 0.05 and *post-hoc* comparisons for the force, EMG and RPE data were evaluated using Bonferroni corrected *t*-tests. Given the fair number of multiple comparisons that were made for the fNIRS data, FDR corrections with *q* = 0.1 were conducted on ΔHbO to reduce the chance of type-1 error.

## Results

Table 1 lists participant demographics and PA outcomes. While significant sex differences in anthropometric measurements were found as expected, males and females were comparable in their PA levels. Finally, while both groups reported similar levels of cognitive, physical, and mental functioning, males reported higher emotional well-being than females.

### Strength, Fatigue, and Perceived Exertion

**Figure 3:**
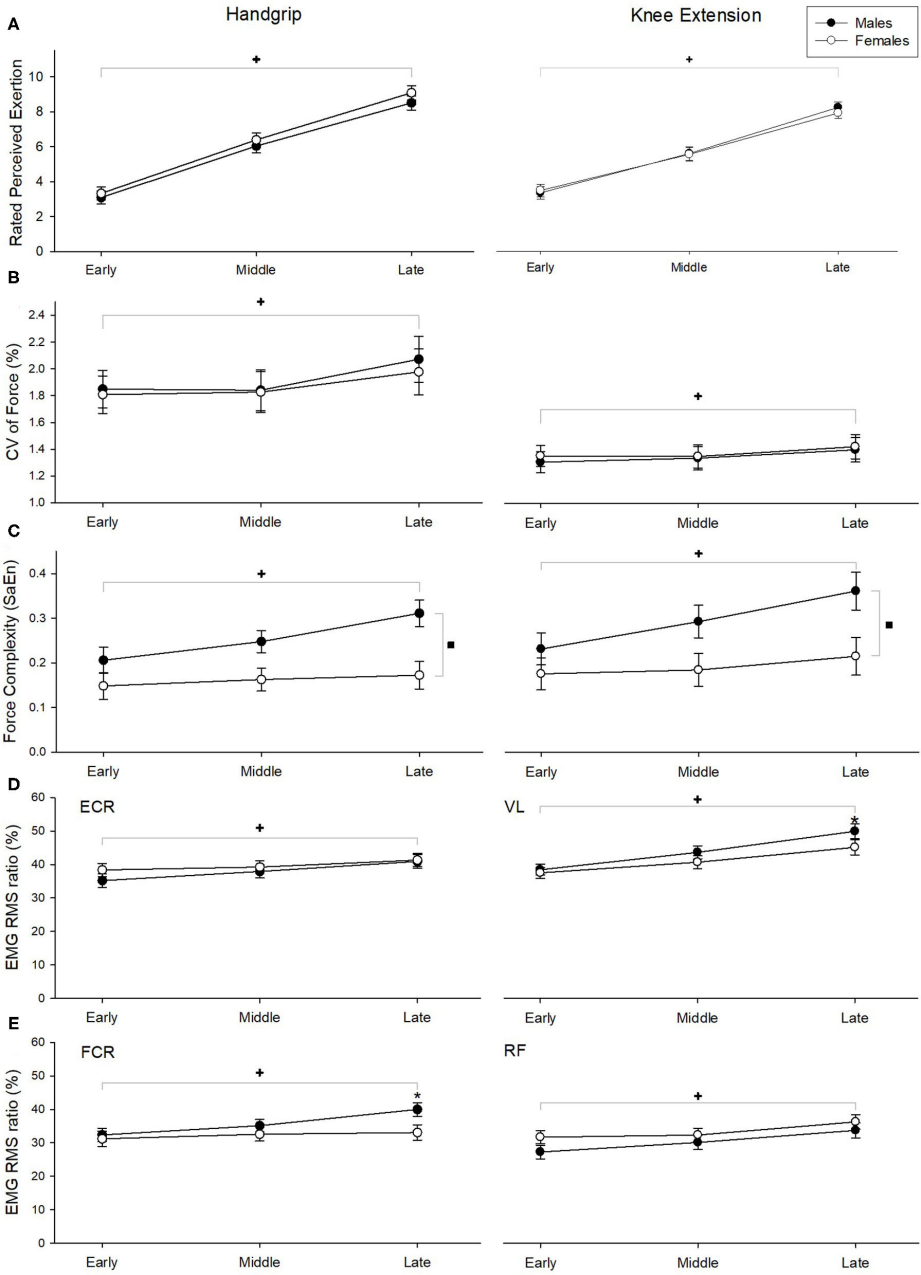
Ratings of perceived exertion **(A)**, CV of force **(B)**, SaEn of force **(C)**, and EMG RMS ratio **(D, E)** during handgrip exercises (left column) and knee extension exercises (right column). ^+^Indicate significant fatigue main effect, ^▪^indicate significant sex main effect, and *indicate sex effects at the specific fatigue phase (fatigue x sex interaction). Statistical significance was determined at *p* < 0.05. Error bars represent standard error.

Compared to females, males exhibited greater initial handgrip and knee extension strength (handgrip: t_(28)_ = 2.348, *p* = 0.026 knee extension: t_(28)_ = 4.12, *p* < 0.001). While strength loss remained comparable between males and females during the handgrip exercise (*p* = 0.37), males lost more strength than females during the knee extension exercise; t_(28)_ = 3.08, *p* = 0.005. Similarly, while endurance time for the handgrip exercise were similar between males and females (*p* = 0.806), males exhibited longer endurance times than females during the knee extension exercise; t_(28)_ = 0.282, *p* = 0.009. Mean (SD) of the strength and fatigue indicators are presented in Table 2. RPE increased significantly over time during both handgrip (handgrip fatigue main effect: F_(2, 56)_ = 215.25, *p* < 0.0001) and knee extension (knee fatigue main effect: F_(2, 56)_ = 243.35, *p* < 0.0001) exercises (Figure 3A). However, there were no sex or sex x fatigue interaction effects observed (both *p*'s > 0.39).

**Table 2:** Strength and fatigue indicators for males and females during handgrip and knee extension exercises.

	**Handgrip**		**Knee extension**	
	**Male**	**Female**	**Male**	**Female**
Initial strength	113.76 (28.44) N	91.2 (22.56) N	146.29 (41.49) Nm	98.16 (18.44) Nm
Strength loss	37.7 (10.2) %	33.8 (12.9) %	41.6 (12.3) %	28.5 (11) %
Endurance time	20.7 (2.3) min	19.9 (2.7) min	27.0 (3.4) min	16.7 (1.4) min

*Values are presented as Mean (SD)*.

### Force Variability

During the handgrip exercise, a main effect of fatigue (F_(2, 56)_ = 10.302, *p* < 0.001; Figure 3B) was found on CV of force, while no sex main effect or fatigue ^*^ sex interaction effect were found (both *p* > 0.8). *Post-hoc* analysis revealed that force fluctuations were greater during late phase than early and middle phases (*p* = 0.01). Both fatigue (F_(2, 56)_ = 11.07, *p* < 0.001) and sex (F_(1, 27)_ = 14.79, *p* = 0.001) significantly affected SaEn, with increasing entropy observed as fatigue developed between early and middle phase (*post-hoc p*-value = 0.01) and greater entropy noted in men vs. women (Figure 3C), with no fatigue ^*^ sex interactions observed (*p* = 0.304).

During the knee extension exercise, CV of torque was significantly greater during the late phase than the early and middle fatigue phases (F_(2, 56)_ = 4.597, *p* = 0.014; Figure 3B), but no differences were seen with sex main effect or fatigue ^*^ sex interaction effect (both *p* > 0.65). SaEn of force was found to be higher during the late when compared to the early and middle fatigue phases (fatigue main effect: F_(2, 56)_ = 8.96, *p* < 0.0001) and higher in males than females (sex main effect: F_(1, 27)_ = 6.76, *p* = 0.015). A significant fatigue ^*^ sex interaction effect (F_(2, 56)_ = 3.62, *p* = 0.033) was also found; while SaEn was higher in the late when compared to the early phase for males (*post-hoc p*-value < 0.01), it remained comparable across the fatigue phases for females (Figure 3C).

### Electromyography

EMG RMS of the respective muscles (Figures 3D,E) increased significantly over time for both handgrip (ECR: F_(2,56)_ = 16.365, *p* < 0.001, FCR: F_(2,56)_ = 22.737, *p* < 0.001) and knee extension (VL: F_(2,56)_ = 57.924, *p* < 0.001, RF: F_(2,56)_ = 49.379, *p* < 0.001) exercises. A significant fatigue ^*^ sex interaction effect was found on the FCR EMG RMS (F_(2,56)_ = 9.002, *p* = 0.001); males exhibited higher EMG RMS than females at the late fatigue phase (all *post-hoc p*-values < 0.02). However, changes in ECR EMG RMS during handgrip exercise and VL and RF EMG RMS during knee extension were comparable between males and females (all *p*'s > 0.27).

### fNIRS

**Figure 4:**
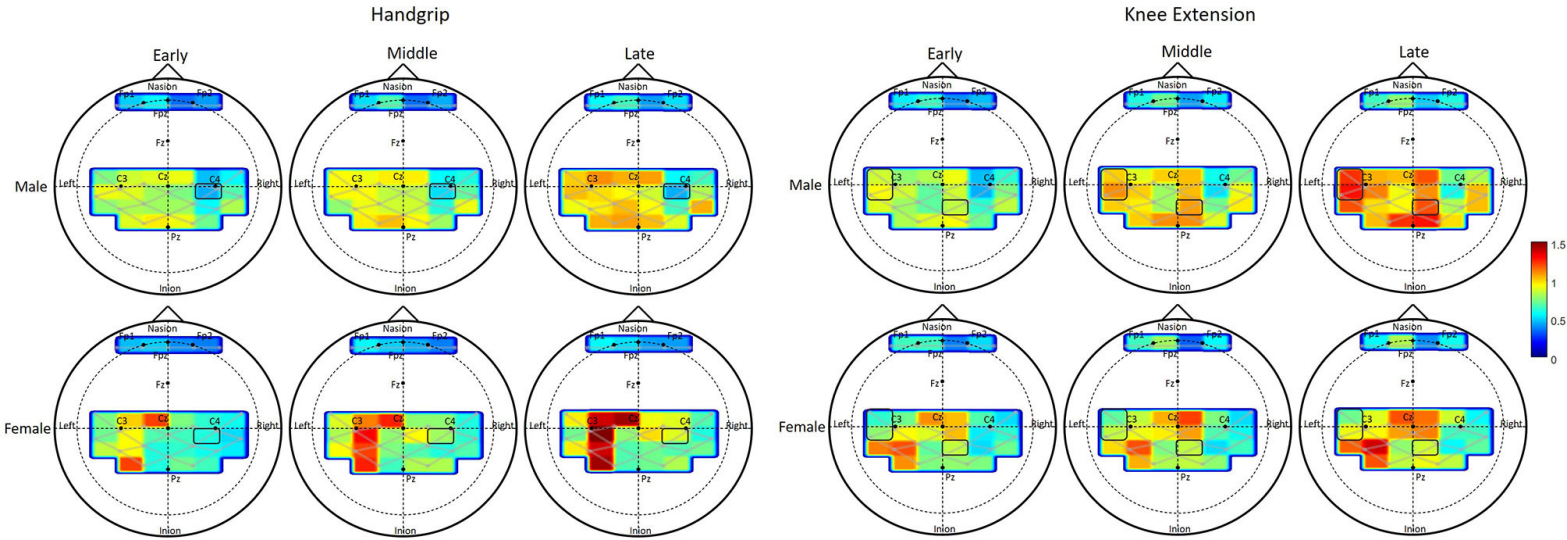
Change of oxygenated blood flow (ΔHbO) during fatigue development. Each column shows the averaged HbO activation within each phase, early, middle, and late. Top two rows show the HbO activation changes of male and females during handgrip exercise, and bottom two rows show that during knee extension exercise. Each activation map shows the HbO activation of each channel for each group in each phase. Level of activation is displayed as graded color shown in the right side of the figure.

Task related oxygenated hemoglobin changes in cerebral blood flow (ΔHbO, respectively) during handgrip exercise increased with fatigue development at 13 channels (refer to Table 3 for *p*-values). ΔHbO change across fatigue development was greater for females than males at channel 6 monitoring the right motor region (Figure 4). Males showed overall greater ΔHbO than females at the left sensory area (sex main effect in channel 14).

**Table 3:** Observed p-values for each channel of fNIRS measurements.

			**Handgrip**			**Knee extension**		
**Area**	**Hemisphere**	**Channel**	**Fatigue**	**F x G**	**Sex**	**Fatigue**	**F x G**	**Sex**
Frontal	Right	Ch1	0.473	0.556	0.154	0.044	0.939	0.866
		Ch2	0.147	0.648	0.896	0.742	0.063	0.881
	Left	Ch16	0.029	0.604	0.345	0.002^*^	0.564	0.829
		Ch17	0.103	0.276	0.518	0.024	0.223	0.333
Motor	Right	Ch27	0.001^*^	0.983	0.194	0.017	0.224	0.548
		Ch28	<0.001^*^	0.109	0.702	0.046	0.456	0.609
		Ch5	<0.001^*^	0.152	0.051	<0.001^*^	0.553	0.127
		Ch6	<0.001^*^	<0.001^*^	0.033	0.005^*^	0.563	0.316
		Ch3	0.049	0.944	0.752	0.019	0.174	0.029
		Ch4	0.048	0.388	0.112	0.003^*^	0.015	0.013
	Left	Ch29	<0.001^*^	0.105	0.571	0.013	0.953	0.538
		Ch30	0.028	0.382	0.265	0.002^*^	0.967	0.230
		Ch20	0.001^*^	0.672	0.246	<0.001^*^	0.375	0.395
		Ch21	0.001^*^	0.017	0.050	0.094	0.131	0.505
		Ch18	0.002^*^	0.821	0.875	<0.001^*^	0.008^*^	0.023
		Ch19	0.007^*^	0.533	0.936	<0.001^*^	0.007^*^	0.060
Sensory	Right	Ch12	0.047	0.720	0.057	<0.001^*^	0.001^*^	0.150
		Ch13	0.378	0.269	0.071	<0.001^*^	0.100	0.085
		Ch9	0.033	0.377	0.283	0.008^*^	0.314	0.901
		Ch10	0.009^*^	0.704	0.811	0.012	0.213	0.643
		Ch8	0.002^*^	0.577	0.051	0.189	0.103	0.072
	Left	Ch15	0.001^*^	0.042	0.025	0.001^*^	0.137	0.613
		Ch14	0.075	0.702	0.003^*^	0.001^*^	0.062	0.062
		Ch23	0.102	0.833	0.141	0.062	0.270	0.421
		Ch24	0.027	0.450	0.513	0.002^*^	0.043	0.758
		Ch25	<0.001^*^	0.071	0.097	0.009^*^	0.306	0.070

* *Indicates statistical significance with FDR corrections with q = 0.1. The order of channels is based on spatial location of the channel on the brain surface, anterior to posterior, and medial to lateral*.

ΔHbO during knee extension exercise increased with fatigue development at a part of the right frontal area and at a part of the motor and sensory area of both hemispheres (fatigue main effect in 15 channels, see Table 3). The amount of ΔHbO change with fatigue development was greater with males than females in the right sensory area (channel 12) and left motor area (channels 18, 19, see Table 3).

## Discussion

In general, increasing fatigability, i.e., increases in force fluctuations and sample entropy, was associated with changes in neural activation across several brain regions, independent of the muscle groups. During handgrip fatigue, increased activation of the contralateral motor area (channels 18, 19, 20, 21, and 29), ipsilateral motor area (channels 5, 6, 27, and 28), contralateral sensory area (channels 8 and 10), and ipsilateral sensory area (channels 15 and 25) were observed. Interestingly, fatigue-related increases in prefrontal activation during handgrip fatigue were found to have marginal effects, with no statistical significance, however the trends were found to be similar to those reported by Liu et al. (2003) in a study that examined changes in brain function during handgrip fatigue in young adults. During knee extension fatigue, brain activation increased with fatigue development at contralateral frontal area (channel 1), contralateral motor area (channels 18, 29, 20, 30), ipsilateral motor area (channels 4, 5, 6, 27), contralateral sensory area (channels 14, 15, 24, 25), and ipsilateral sensory area (channels 9, 10, 12, 13). The spatial location of areas that showed increased activation matches with the previous results reported on cortical areas responsible for lower extremity muscle movement, such as bilateral medial motor and sensory areas (Dobkin et al., 2004; Hollnagel et al., 2011). Increasing engagement of the contralateral motor and sensory areas suggest that fatigue manifestation increases the need for altering the ongoing descending commands based on fatigue-related analyzed sensory information (Liu et al., 2003). Additionally, while the spatial activation pattern within each phase of fatigue was not examined in the present study, the patterns appear to have strong left lateralization (see Figure 4) during handgrip when compared to knee exercises, similar to that reported in fMRI investigations of adults aged 28–54 years (Luft et al., 2002). The present study also observed concomitant increases in muscle activation over time across the upper and lower extremity muscles. These fatigue-related changes in neural and peripheral measures are similar to other studies that used different hemodynamic neuroimaging methods, such as fMRI and PET (Dai et al., 2001; van Duinen et al., 2008).

During the handgrip exercises, males exhibited stronger maximum voluntary contraction force (i.e., initial strength) than females, however endurance time and strength loss after fatigue development were comparable between groups. These findings are consistent with results from prior studies (Gonzales and Scheuermann, 2007; Jarvis et al., 2011). Perceived fatigue levels increased similarly over time across both sexes. While force fluctuations were comparable across males and females, force complexity, measured using sample entropy of force, was in general higher in males than females (i.e., sex main effect). Force complexity provides information on system adaptability, particularly as it relates to amplitude of force steadiness, with less complex signals generating more regular fluctuations (Lipsitz and Goldberger, 1992). Loss in complexity in physiological time series when accompanied with increased fluctuations may be associated with inability of the motor system to remain steady or extend time to task failure, particularly during maximal fatiguing exercises, in young adults (Pethick et al., 2015). However, the present study observed increasing force complexity over time (i.e., fatigue main effect), similar to that found by Svendsen and Madeleine (2010). It is likely that the force contraction levels, 20% MVC in Svendsen and Madeleine (2010) and 30% in the present study compared to maximal contractions in Pethick et al. (2015), may have accounted for the differences in forces complexity trends with fatigue. In the present study, higher complexity of force time series, in combination with increased FCR EMG activity may be indicative of increased system adaptability in older males than older females (Srinivasan and Mathiassen, 2012). Males also exhibited increased activation in one channel within the sensory motor region of the left hemisphere than females. It is likely that the significant increase in the activation of ipsilateral motor regions, observed in females at the late fatigue phase, was an effort to increase central neural drive in response to the loss of force generating capacity (Hunter et al., 2009; Burnley et al., 2012). These findings implicate sex differences in neuromuscular strategies in older adults during handgrip fatigue exercises. Similar compensatory ipsilateral cortical activation was observed in females than males using fMRI, with comparable motor performance, during finger tapping tasks among young adults (Lissek et al., 2007).

During the knee extensor exercises, males exhibited greater initial strength than females, a finding that is widely established (Pincivero et al., 2003). However, unlike our handgrip fatigue results, longer endurance times and greater strength loss was observed in males than females. These findings deviate from the larger literature on sex differences in knee extensor fatigability (Clark et al., 2005; Ansdell et al., 2017), but are supported by few others (Pincivero et al., 2003). Perceived effort, force fluctuation and complexity increased over time, as was expected, in response to the fatigue development along with increases in muscle activity of the vastus lateralis and rectus femoris muscles. However, force complexity trends were different across sexes during knee extensor fatigue. While force complexity level of older males was increased over time and was greater than females, force fluctuation profiles was not different between the two sexes. It is to be noted that the endurance times were significantly longer for males than females, and thus it is likely that to exhibit the magnitude of motor steadiness (i.e., force fluctuation) comparable to their female counterparts, but lasting longer, males had to often correct their force outputs (Vaillancourt et al., 2003). The concurrent activation increases in the contralateral motor regions (channels 18, 19) and ipsilateral sensory area (channel 12) seen in males over time may implicate an effective neuromotor strategy adopted by older males to maintain performance over time that slows the rate of fatigue development. While no firm reasons can be offered to explain why increased activation in the contralateral motor regions in males were observed, prior research has reported the increased engagement of the ipsilateral sensorimotor cortex to compensate for fatigue-related alteration in motor control and to maintain optimal descending output (Liu et al., 2002, 2003, 2007).

The concurrent assessment of neural dynamics and motor strategies during fatiguing exercises of muscle groups that are both structurally and functionally different provided unique insights to better understanding the mechanisms of neuromuscular fatigue and associated sex differences. Prior neuroimaging studies that examined sex differences during motor control found different activation strategies during upper extremity exercises (Lissek et al., 2007) but not lower extremity exercises (Yoon et al., 2014). It should be noted that the lack of knowledge on sex differences in brain dynamics during neuromuscular fatigue development across upper and lower extremity muscles, which the present study aimed to address, better elucidated sex differences in neuromotor strategies during the handgrip exercise than knee extensor exercises. These is consensus in the literature on the absence of sex differences in muscle fatiguability of the distal upper extremities (Hunter, 2009), but the fNIRS findings here identify the differences in the underlying neural strategies adopted by males and females in maintaining downstream motor outputs. In particular, the increased ipsilateral cortical activation in older females highlight the added cost of preserving task performance during fatigue, which otherwise goes unnoticed when comparing physical demands and capabilities between sexes for work and activities of daily living. In a similar vein, increases in neural activation patterns (i.e., concurrent increase in motor and sensory areas) in males during knee exercises over time point to potential rehabilitation/exercise strategies that may be targeted to benefit more fatigable older individuals to improve neuromotor outcomes.

There are some limitations that exist in the present study. First, the examined relationship between neuromuscular functionality and brain activation is only correlational. In other words, neuromuscular functions changed with fatigue development and so as the brain activation, however the current findings do not provide any causal inference between the two. Future work should extend the current findings by measuring motor evoked potential and active motor threshold using transcranial magnetic stimulation during fatiguing motor tasks (Todd et al., 2003) to confirm sex differences in neural mechanisms of motor fatigue. However, the present study identifies key candidate neural sites that may be targeted in subsequent causal studies to more robustly quantify the mechanistic pathways of fatigability across sexes. Second, the tasks employed in this study are static in nature, and while both handgrip and knee functioning have clinical relevance in older adults (Singh et al., 2010), studies that map cortical activation during more dynamic fatiguing exercises are warranted. Third, the fatiguing protocol was performed at a set pace, using visual cues, of 15 s of exertion followed by 15 s of rest, until voluntary exhaustion. It is well-known that a set pace of work and rest may create confounding human responses, such as anticipation, that may impact neural responses of motor preparation and planning processes (Zhu et al., 2020). However, randomly changing rest times can impact motor fatigue development and reduce its generalizability to available fatigue models (Rashedi and Nussbaum, 2015). Fourth, it is likely that task repetition may result in distinct brain activation patterns that reflect habituation that were not delineated in the present study. Prior studies have addressed such changes to including a control task, i.e., tasks conducted with similar muscle activation but at different intensities (Fry et al., 2017). However, the main purpose of the study was to examine sex differences during fatigability, which in the present analyses, may also be reflective of sex differences in task habituation. Fifth, our study utilized a more liberal approach to correcting for multiple comparisons with the brain data (i.e., *q* = 0.1) to examine the interaction between sex and fatigue phases. It is likely that more targeted monitoring of focused brain regions, based on selection of key ROIs observed in this study, in future work or recruiting a larger study sample may address this limitation. Finally, fNIRS provides a cost-effective and posture-feasible alternative approach to monitoring cortical activation during strenuous motor tasks. However, fatigue-related changes in brain function also include non-cortical regions that can only be recorded through the use of PET or fMRI (van Duinen et al., 2007; DeLuca et al., 2009). However, findings obtained here on cortical regions engaged during handgrip fatigue, a protocol feasible with fMRI, are corroborated with existing fMRI studies (Lissek et al., 2007), thus increasing the confidence of fNIRS as a valuable brain imaging technique to understand neural control of motor fatigue.

## Conclusion

The present study investigated sex differences in spatiotemporal neural activation changes during isometric fatiguing contractions in older adults. To this end, we utilized a neuroergonomics approach, by employing functional near infrared spectroscopy, to monitor neural activation patterns during handgrip and knee extension fatiguing exercises. Important results found in this study include sex differences in fatigability during knee exercises but not during handgrip exercises. Older males exhibited an effective neuromotor strategy to delay knee extensor exhaustion via concomitant motor corrections and cortical activation. While the extent of handgrip fatigue was comparable between males and females, there were significant sex differences in motor cortical activation patterns suggesting different neural strategies adopted by males and females under fatigue to maintain motor performance. A neuroergonomics approach that concurrently assesses neural dynamics and motor strategies during upper and lower extremity motor fatigue provides unique insights on the mechanisms of neuromuscular fatigue and reveals associated sex differences.

## Data Availability Statement

The raw data supporting the conclusions of this article will be made available by the authors, without undue reservation.

## Ethics Statement

The studies involving human participants were reviewed and approved by Institutional Review Board at Texas A&M University. The patients/participants provided their written informed consent to participate in this study.

## Author Contributions

RM conceptualized the study and supervised and guided the data collection. JR performed the data collection and analyzed the data. RM and JR synthesized the results and drafted the manuscript. All authors contributed to the article and approved the submitted version.

## Author Disclaimer

The content is solely the responsibility of the authors and does not necessarily represent the official views of the National Institutes of Health.

## Conflict of Interest

The authors declare that the research was conducted in the absence of any commercial or financial relationships that could be construed as a potential conflict of interest.

## Publisher's Note

All claims expressed in this article are solely those of the authors and do not necessarily represent those of their affiliated organizations, or those of the publisher, the editors and the reviewers. Any product that may be evaluated in this article, or claim that may be made by its manufacturer, is not guaranteed or endorsed by the publisher.

## References

[B1] Abdel-Malek D. M. Foley R. C. Wakeely F. Graham J. D. La Delfa N. J. J. H. F. (2020). Exploring localized muscle fatigue responses at current upper-extremity ergonomics threshold limit values. Hum. Factors. 0018720820940536. 10.1177/0018720820940536 32757794

[B2] Ansdell P. Thomas K. Howatson G. Hunter S. K. Goodall S. (2017). Contraction intensity and sex differences in knee-extensor fatigability. J. Electromyogr. Kinesiol. 37, 68–74. 10.1016/j.jelekin.2017.09.003 28963937

[B3] Avin K. G. Naughton M. R. Ford B. W. Moore H. E. Monitto-Webber M. N. Stark A. M.. (2010). Sex differences in fatigue resistance are muscle group dependent. Med. Sci. Sports Exerc. 42, 1943–1950. 10.1249/MSS.0b013e3181d8f8fa 20195184 PMC2917609

[B4] Berrios G. E. (1990). Feelings of fatigue and psychopathology: a conceptual history. Compr. Psychiatry 31, 140–151. 10.1016/0010-440X(90)90018-N 2178863

[B5] Borg G. Hassmén P. Lagerström M. (1987). Perceived exertion related to heart rate and blood lactate during arm and leg exercise. Eur. J. Appl. Physiol. Occup. Physiol. 56, 679–685. 10.1007/BF00424810 3678222

[B6] Burnley M. Vanhatalo A. Jones A. M. (2012). Distinct profiles of neuromuscular fatigue during muscle contractions below and above the critical torque in humans. J. Appl. Physiol. J. Appl. Physiol. 113, 215–223. 10.1152/japplphysiol.00022.2012 22556396

[B7] Chiarelli A. M. Maclin E. L. Fabiani M. Gratton G. (2015). A kurtosis-based wavelet algorithm for motion artifact correction of fNIRS data. NeuroImage 112, 128–137. 10.1016/j.neuroimage.2015.02.057 25747916 PMC4408240

[B8] Clark B. C. Collier S. R. Manini T. M. Ploutz-Snyder L. L. (2005). Sex differences in muscle fatigability and activation patterns of the human quadriceps femoris. Eur. J. Appl. Physiol. 94, 196–206. 10.1007/s00421-004-1293-0 15791418

[B9] Dai T. H. Liu J. Z. Sahgal V. Brown R. W. Yue G. H. (2001). Relationship between muscle output and functional MRI-measured brain activation. Exp. Brain Res. 140, 290–300. 10.1007/s002210100815 11681304

[B10] de Rekeneire N. Leo-Summers L. Han L. Gill T. M. (2014). Epidemiology of restricting fatigue in older adults: the precipitating events project. J. Am. Geriatr. Soc. 62, 476–481. 10.1111/jgs.12685 24512073 PMC4103720

[B11] DeLuca J. Genova H. M. Capili E. J. Wylie G. R. (2009). Functional neuroimaging of fatigue. Phys. Med. Rehabil. Clin. N. Am. 20, 325–337. 10.1016/j.pmr.2008.12.007 19389614

[B12] Dobkin B. H. Firestine A. West M. Saremi K. Woods R. (2004). Ankle dorsiflexion as an fMRI paradigm to assay motor control for walking during rehabilitation. NeuroImage 23, 370–381. 10.1016/j.neuroimage.2004.06.008 15325385 PMC4164211

[B13] Enoka R. M. Christou E. A. Hunter S. K. Kornatz K. W. Semmler J. G. Taylor A. M.. (2003). Mechanisms that contribute to differences in motor performance between young and old adults. J. Electromyogr. Kinesiol. 13, 1–12. 10.1016/S1050-6411(02)00084-6 12488083

[B14] Farina D. Holobar A. Merletti R. Enoka R. M. (2010). Decoding the neural drive to muscles from the surface electromyogram. Clin. Neurophysiol. 121, 1616–1623. 10.1016/j.clinph.2009.10.040 20444646

[B15] Fry A. Mullinger K. J. O'Neill G. C. Brookes M. J. Folland J. P. (2017). The effect of physical fatigue on oscillatory dynamics of the sensorimotor cortex. Acta Physiol. (Oxf) 220, 370–381. 10.1111/apha.12843 27981752

[B16] Gonzales J. U. Scheuermann B. W. (2007). Absence of gender differences in the fatigability of the forearm muscles during intermittent isometric handgrip exercise. J. Sports Sci. Med. 6, 98–105. 24149231 PMC3778706

[B17] Good C. D. Johnsrude I. Ashburner J. Henson R. N. Friston K. J. Frackowiak R. S. (2001). Cerebral asymmetry and the effects of sex and handedness on brain structure: a voxel-based morphometric analysis of 465 normal adult human brains. NeuroImage 14, 685–700. 10.1006/nimg.2001.0857 11506541

[B18] Grooms D. R. Page S. J. Onate J. A. (2015). Brain activation for knee movement measured days before second anterior cruciate ligament injury: neuroimaging in musculoskeletal medicine. J. Athl. Train. 50, 1005–1010. 10.4085/1062-6050-50.10.02 26509775 PMC4641538

[B19] Hollnagel C. Brügger M. Vallery H. Wolf P. Dietz V. Kollias S.. (2011). Brain activity during stepping: a novel MRI-compatible device. J. Neurosci. Methods 201, 124–130. 10.1016/j.jneumeth.2011.07.022 21827788

[B20] Hunter S. K. (2009). Sex differences and mechanisms of task-specific muscle fatigue. Exerc. Sport Sci. Rev. 37, 113–122. 10.1097/JES.0b013e3181aa63e2 19550202 PMC2909485

[B21] Hunter S. K. (2014). Sex differences in human fatigability: mechanisms and insight to physiological responses. Acta Physiol. 210, 768–789. 10.1111/apha.12234 24433272 PMC4111134

[B22] Hunter S. K. (2016). The relevance of sex differences in performance fatigability. Med. Sci. Sports Exerc. 48, 2247–2256. 10.1249/MSS.0000000000000928 27015385 PMC5349856

[B23] Hunter S. K. Butler J. E. Todd G. Gandevia S. C. Taylor J. L. (2006). Supraspinal fatigue does not explain the sex difference in muscle fatigue of maximal contractions. J. Appl. Physiol. 101, 1036–1044. 10.1152/japplphysiol.00103.2006 16728525

[B24] Hunter S. K. Enoka R. M. (2001). Sex differences in the fatigability of arm muscles depends on absolute force during isometric contractions. J. Appl. Physiol. 91, 2686–2694. 10.1152/jappl.2001.91.6.2686 11717235

[B25] Hunter S. K. Griffith E. E. Schlachter K. M. Kufahl T. D. (2009). Sex differences in time to task failure and blood flow for an intermittent isometric fatiguing contraction. Muscle Nerve 39, 42–53. 10.1002/mus.21203 19086076

[B26] Ingalhalikar M. Smith A. Parker D. Satterthwaite T. D. Elliott M. a.. (2014). Sex differences in the structural connectome of the human brain. Proc. Natl. Acad. Sci. U.S.A. 111, 823–828. 10.1073/pnas.1316909110 24297904 PMC3896179

[B27] Iridiastadi H. Nussbaum M. A. J. H. f. (2006). Muscular fatigue and endurance during intermittent static efforts: effects of contraction level, duty cycle, and cycle time. Hum. Factors 48, 710–720. 10.1518/001872006779166389 17240719

[B28] Jarvis S. S. VanGundy T. B. Galbreath M. M. Shibata S. Okazaki K. Reelick M. F.. (2011). Sex differences in the modulation of vasomotor sympathetic outflow during static handgrip exercise in healthy young humans. Am. J. Physiol. Regul. Integr. Comp. Physiol. 301, R193–R200. 10.1152/ajpregu.00562.2010 21508291 PMC3129874

[B29] Karim H. Schmidt B. Dart D. Beluk N. Huppert T. (2012). Functional near-infrared spectroscopy (fNIRS) of brain function during active balancing using a video game system. Gait Posture 35, 367–372. 10.1016/j.gaitpost.2011.10.007 22078300 PMC3294084

[B30] Keisker B. Hepp-Reymond M. C. Blickenstorfer A. Meyer M. Kollias S. S. (2009). Differential force scaling of fine-graded power grip force in the sensorimotor network. Hum. Brain Mapp. 30, 2453–2465. 10.1002/hbm.20676 19172654 PMC6871245

[B31] Kennel M. B. Brown R. Abarbanel H. D. (1992). Determining embedding dimension for phase-space reconstruction using a geometrical construction. J. Phys. Rev. A 45:3403. 10.1103/PhysRevA.45.3403 9907388

[B32] Koenraadt K. L. M. Roelofsen E. G. J. Duysens J. Keijsers N. l. L. W. (2014). Cortical control of normal gait and precision stepping: an fNIRS study. NeuroImage 85, 415–422. 10.1016/j.neuroimage.2013.04.070 23631980

[B33] Lewis G. Wessely S. (1992). The epidemiology of fatigue: more questions than answers. J. Epidemiol. Community Health 46, 92–97. 10.1136/jech.46.2.92 1583440 PMC1059513

[B34] Lipsitz L. A. Goldberger A. L. (1992). Loss of'complexity'and aging: potential applications of fractals and chaos theory to senescence. J. JAMA 267, 1806–1809. 10.1001/jama.1992.03480130122036 1482430

[B35] Lissek S. Hausmann M. Knossalla F. Peters S. Nicolas V. Güntürkün O.. (2007). Sex differences in cortical and subcortical recruitment during simple and complex motor control: an fMRI study. NeuroImage 37, 912–926. 10.1016/j.neuroimage.2007.05.037 17629502

[B36] Liu J. Z. Dai T. H. Sahgal V. Brown R. W. Yue G. H. (2002). Nonlinear cortical modulation of muscle fatigue: a functional MRI study. Brain Res 957, 320–329. 10.1016/S0006-8993(02)03665-X 12445974

[B37] Liu J. Z. Lewandowski B. Karakasis C. Yao B. Siemionow V. Sahgal V.. (2007). Shifting of activation center in the brain during muscle fatigue: an explanation of minimal central fatigue? NeuroImage 35, 299–307. 10.1016/j.neuroimage.2006.09.050 17236789 PMC2701907

[B38] Liu J. Z. Shan Z. Y. Zhang L. D. Sahgal V. Brown R. W. Yue G. H. (2003). Human brain activation during sustained and intermittent submaximal fatigue muscle contractions: an FMRI study. J. Neurophysiol. 90, 300–312. 10.1152/jn.00821.2002 12634278

[B39] Luft A. R. Smith G. V. Forrester L. Whitall J. Macko R. F. Hauser T.-K.. (2002). Comparing brain activation associated with isolated upper and lower limb movement across corresponding joints. Hum. Brain Mapp. 17, 131–140. 10.1002/hbm.10058 12353246 PMC6872124

[B40] Malonek D. Grinvald A. (1996). Interactions between electrical activity and cortical microcirculation revealed by imaging spectroscopy: implications for functional brain mapping. Science 272, 551–554. 10.1126/science.272.5261.551 8614805

[B41] Mantooth W. P. Mehta R. K. Rhee J. Cavuoto L. A. (2018). Task and sex differences in muscle oxygenation during handgrip fatigue development. Ergonomics 61, 1646–1656. 10.1080/00140139.2018.1504991 30317942

[B42] Martin P. G. Rattey J. (2007). Central fatigue explains sex differences in muscle fatigue and contralateral cross-over effects of maximal contractions. Pflugers Arch. 454, 957–969. 10.1007/s00424-007-0243-1 17342531

[B43] Maughan R. Harmon M. Leiper J. Sale D. Delman A. (1986). Endurance capacity of untrained males and females in isometric and dynamic muscular contractions. Eur. J. Appl. Physiol. Occup. Physiol. 55, 395–400. 10.1007/BF00422739 3758040

[B44] Mehta R. K. Agnew M. J. (2008). An Investigation of the Impact of Fatigue and Aging on the Performance of Spatially Constrained Assembly Tasks Commonly Found in the Construction Industry. Proc. Hum. Factors Ergon. Soc. Annu. Meet. 52, 1795–1799. 10.1177/154193120805202203

[B45] Mehta R. K. Rhee J. (2017). Age-specific neural strategies to maintain motor performance after an acute social stress bout. Exp. Brain Res. 235, 2049–2057. 10.1007/s00221-017-4949-9 28357463

[B46] Mehta R. K. Shortz A. E. (2014). Obesity-related differences in neural correlates of force control. Eur. J. Appl. Physiol. 114, 197–204. 10.1007/s00421-013-2762-0 24197082

[B47] Pethick J. Winter S. L. Burnley M. (2015). Fatigue reduces the complexity of knee extensor torque fluctuations during maximal and submaximal intermittent isometric contractions in man. J. Physiol. 593, 2085–2096. 10.1113/jphysiol.2015.284380 25664928 PMC4405761

[B48] Pethick J. Winter S. L. Burnley M. (2019). Fatigue reduces the complexity of knee extensor torque during fatiguing sustained isometric contractions. Eur. J. Sport Sci. 19, 1–10. 10.1080/17461391.2019.1599450 30955469

[B49] Pincivero D. M. Gandaio C. M. Ito Y. (2003). Gender-specific knee extensor torque, flexor torque, and muscle fatigue responses during maximal effort contractions. Eur J. Appl. Physiol. 89, 134–141. 10.1007/s00421-002-0739-5 12665976

[B50] Quaresima V. Ferrari M. (2016). Functional Near-Infrared Spectroscopy (fNIRS) for assessing cerebral cortex function during human behavior in natural/social situations: a concise review. Organ. Res. Methods 22, 1–23. 10.1177/1094428116658959

[B51] Rashedi E. Nussbaum M. A. (2015). A review of occupationally–relevant models of localised muscle fatigue. Int. J. Hum.Factors Model. Simul. 5, 61–80. 10.1504/IJHFMS.2015.068119 PMC4681880 26656741

[B52] Rhee J. Mehta R. K. (2019). Quantifying brain hemodynamics during neuromuscular fatigue, in Neuroergonomics, eds Hasan A. Dehais F. (Amsterdam: Elsevier), 175–180. 10.1016/B978-0-12-811926-6.00029-4

[B53] Richman J. S. Moorman J. R. (2000). Physiological time-series analysis using approximate entropy and sample entropy. Am. J. Physiol. Heart Circ. Physiol. 278, H2039–H2049. 10.1152/ajpheart.2000.278.6.H2039 10843903

[B54] Sacher J. Neumann J. Okon-Singer H. Gotowiec S. Villringer A. (2013). Sexual dimorphism in the human brain: evidence from neuroimaging. Magn. Reson. Imaging 31, 366–375. 10.1016/j.mri.2012.06.007 22921939

[B55] Samani A. Srinivasan D. Mathiassen S. E. Madeleine P. J. Kinesiology (2015). Nonlinear metrics assessing motor variability in a standardized pipetting task: between-and within-subject variance components. J. Electrimyogr. Kinesiol. 25, 557–564. 10.1016/j.jelekin.2015.01.005 25681167

[B56] Scholkmann F. Spichtig S. Muehlemann T. Wolf M. (2010). How to detect and reduce movement artifacts in near-infrared imaging using moving standard deviation and spline interpolation. Physiol. Meas. 31, 649. 10.1088/0967-3334/31/5/004 20308772

[B57] Singh N. B. Arampatzis A. Duda G. Heller M. O. Taylor W. R. (2010). Effect of fatigue on force fluctuations in knee extensors in young adults. Philos. Transact. A. Math. Phys. Eng. Sci. 368, 2783–2798. 10.1098/rsta.2010.0091 20439273

[B58] Srinivasan D. Mathiassen S. E. (2012). Motor variability in occupational health and performance. J. Clin. Biomech. 27, 979–993. 10.1016/j.clinbiomech.2012.08.007 22954427

[B59] Srinivasan D. Sinden K. E. Mathiassen S. E. Côté J. N. (2016). Gender differences in fatigability and muscle activity responses to a short-cycle repetitive task. Eur. J. Appl. Physiol. 116, 2357–2365. 10.1007/s00421-016-3487-7 27743025 PMC5118407

[B60] Sukal-Moulton T. de Campos A. C. Stanley C. J. Damiano D. L. (2014). Functional near infrared spectroscopy of the sensory and motor brain regions with simultaneous kinematic and EMG monitoring during motor tasks. J. Vis. Exp. 94:52391. 10.3791/52391 25548919 PMC4396965

[B61] Sung P. S. Zurcher U. Kaufman M. (2008). Gender differences in spectral and entropic measures of erector spinae muscle fatigue. J. Rehabil. Res. Dev. 45, 1431–1439. 10.1682/JRRD.2007.11.0196 19319765

[B62] Svendsen J. H. Madeleine P. (2010). Amount and structure of force variability during short, ramp and sustained contractions in males and females. Hum. Mov. Sci. 29, 35–47. 10.1016/j.humov.2009.09.001 19853318

[B63] Taylor J. L. Gandevia S. C. (2008). A comparison of central aspects of fatigue in submaximal and maximal voluntary contractions. J. Appl. Physiol. 104, 542–550. 10.1152/japplphysiol.01053.2007 18032577

[B64] Todd G. Petersen N. T. Taylor J. L. Gandevia S. C. (2003). The effect of a contralateral contraction on maximal voluntary activation and central fatigue in elbow flexor muscles. Exp. Brain Res. 150, 308–313. 10.1007/s00221-003-1379-7 12677313

[B65] Tralongo P. Respini D. Ferraù F. (2003). Fatigue and aging. Crit. Rev. Oncol. Hematol. 48, S57–S64. 10.1016/j.critrevonc.2003.07.003 14563522

[B66] Vaillancourt D. E. Larsson L. Newell K. M. (2003). Effects of aging on force variability, single motor unit discharge patterns, and the structure of 10, 20, and 40 Hz EMG activity. Neurobiol. Aging 24, 25–35. 10.1016/S0197-4580(02)00014-3 12493548

[B67] van Duinen H. Renken R. Maurits N. Zijdewind I. (2007). Effects of motor fatigue on human brain activity, an fMRI study. NeuroImage 35, 1438–1449. 10.1016/j.neuroimage.2007.02.008 17408974

[B68] van Duinen H. Renken R. Maurits N. M. Zijdewind I. (2008). Relation between muscle and brain activity during isometric contractions of the first dorsal interosseus muscle. Hum. Brain Mapp. 29, 281–299. 10.1002/hbm.20388 17394210 PMC6870705

[B69] Yoon T. Schlinder Delap B. Griffith E. E. Hunter S. K. (2007). Mechanisms of fatigue differ after low-and high-force fatiguing contractions in men and women. Muscle Nerve 36, 515–524. 10.1002/mus.20844 17626289

[B70] Yoon T. Vanden Noven M. L. Nielson K. a. Hunter S. K. (2014). Brain areas associated with force steadiness and intensity during isometric ankle dorsiflexion in men and women. Exp. Brain Res. 232, 3133–3145. 10.1007/s00221-014-3976-z 24903120 PMC4172577

[B71] Zhu Y. Rodriguez-Paras C. Rhee J. Mehta R. K. (2020). Methodological approaches and recommendations for functional near-infrared spectroscopy applications in HF/E research. Hum Factors 62, 613–642. 10.1177/0018720819845275 31107601

